# Perinatal Outcome of Singletons Born after Using a Simplified Low-Cost IVF Culture System and All Singletons Born in Flanders (Belgium) between 2012 and 2020

**DOI:** 10.3390/jcm12113683

**Published:** 2023-05-26

**Authors:** Willem Ombelet, Régine Goemaes, Elizaveta Fomenko, Rudi Campo

**Affiliations:** 1 Genk Institute for Fertility Technology, Department of Obstetrics and Gynaecology, ZOL Hospitals, 3600 Genk, Belgium; 2 Faculty of Medicine and Life Sciences, Hasselt University, Agoralaan, 3590 Diepenbeek, Belgium; 3 Centre for Perinatal Epidemiology (SPE), Koning Albert II-laan 35 Box 29, 1030 Brussels, Belgium; regine.goemaes@vlaanderen.be (R.G.); elizaveta.fomenko@vlaanderen.be (E.F.); 4 Life Expert Centre, Schipvaartstraat, 3000 Leuven, Belgium

**Keywords:** assisted reproduction, infertility, IVF, low-cost IVF, low birth weight, natural conception, preterm birth, simplified IVF

## Abstract

Background: We developed a simplified IVF culture system (SCS) which has proven to be effective and safe in a selected IVF cohort. Methods: Preterm birth (PTB) and low birth weight (LBW) of 175 singletons born after using the SCS, 104 after fresh embryo transfer (ET), and 71 after frozen embryo transfer, were compared with all singletons born in Flanders between 2012 and 2020 conceived after natural conception, ovarian stimulation (OS), and assisted reproduction (IVF/ICSI). Findings: The proportion of preterm (<37 weeks) births was significantly higher in the case of IVF or ICSI, followed by hormonal treatment, compared to spontaneous pregnancies. There was no significant difference in PTB between SCS and any of the other groups. Concerning the average birth weight we found no significant difference between singletons born after natural conception and SCS. However, a significant difference in average birth weight was found between SCS singletons and singletons born after IVF, ICSI and hormonal treatment, with a significantly higher birth weight in the SCS group. This difference was also observed in the proportion of babies weighing less than 2500 g, with significantly more LBW babies in the IVF and ICSI group compared to the SCS newborns. Interpretation: Taking into account the small series, PTB and LBW rates in SCS singletons were found to be comparable with singletons born after natural conception. Compared to babies born after ovarian stimulation and IVF/ICSI, SCS singletons had a lower PTB and LBW rates, although the differences were not significant for PTB. Our results confirm previous reports on reassuring perinatal outcomes after using the SCS technology.

## 1. Introduction

With the birth of the first baby conceived by in vitro fertilisation in 1978 [1], the application of IVF to treat human infertility increased exponentially for many years. However, in many areas of the world, continued progress in IVF is not of any value to the vast majority of patients in need because even the most basic elements required to provide IVF are either unavailable or unaffordable [2,3,4,5]. The absence of affordable fertility services in LMIC (Low- and Middle-Income Countries) has been justified by overpopulation and limited resources. This results in inequitable access to infertility treatment compared to developed countries, although the burden of infertility is considerably higher in LMIC with more severe psychosocial, emotional, economic, and health consequences [2]. Therefore, we need low-cost IVF initiatives to simplify IVF-related treatment, reduce costs, and improve access to treatment for individuals in low-resource contexts. The Walking Egg non-profit organisation (npo) was founded in 2010. Right from the start, The Walking Egg npo aimed to make infertility care in all its aspects, including assisted reproductive technologies, available and accessible for a much larger part of the world population. The final objective is the implementation of affordable infertility services in many resource-poor countries [6].

As part of the Walking Egg Project, a simplified, low-cost IVF culture system (SCS) was evolved, which turned out to be less expensive but equally effective and safe when compared to regular IVF and ICSI for a selected group of infertile couples with no severe male factor involved [7].

The effectiveness of this method was studied in a prospective cohort study showing similar ongoing pregnancy and implantation rates for SCS when compared to ICSI followed by regular culturing and using sibling oocytes [8]. In the same study cohort, we found no difference in perinatal outcome between babies born after SCS or ICSI, but the prevalence of preterm birth (PTB) and low birth weight (LBW) in SCS singletons was lower than expected in an IVF or ICSI population [9].

Preterm birth and low birth weight are crucial issues when dealing with neonatal outcomes. The complications of PTB and LBW are leading causes of under-5 child mortality, serious morbidity, and long hospital admissions globally [10,11]. Preterm birth has been associated with an increased risk for neonatal respiratory disorders, necrotising enterocolitis, neurological disorders, and many other medical problems. It also leads to a higher rate of hospital admissions and increasing healthcare costs [12].

Almost all studies describe a worse perinatal outcome after using IVF/ICSI when compared to natural conception, especially after the transfer of fresh embryos. In most studies, the preterm birth rate (PTB) for singletons born after IVF and/or ICSI procedures varies between 8.6 and 14.9%, and low birth weight (LBW) has been reported to vary between 5.9 and 7.0% [13,14,15,16,17,18].

For naturally conceived singletons, a lower prevalence of PTB (5.9–6.4%) and LBW (4.4–5.8%) has been reported [12,13,15].

Several reports show that singletons born after the transfer of frozen embryos have a lower risk for PTB and LBW and a higher risk of being large for their gestational age (LGA) when compared to singletons born after the transfer of fresh embryos [18,19,20]. Compared to naturally conceived newborns, babies born as the result of fresh but not frozen embryo transfer are associated with LBW. Both fresh and frozen are associated with PTB. Frozen is uniquely associated with LGA [19,20,21].

In a recent report and making use of BELRAP data (Belgian Register for Assisted Procreation), we found that for babies born after a fresh embryo transfer (ET), the prevalence of PTB and LBW was significantly lower in SCS singletons when compared to all singletons born after conventional IVF in Belgium during the same study period [22]. Because of this unexpected finding, we decided to compare our SCS singletons with naturally conceived singletons.

Therefore, we compared the prevalence of LBW and PTB of 175 SCS singleton babies born after the transfer of fresh and frozen embryos with perinatal outcome results of all babies born in Flanders, the northern Dutch-speaking part of Belgium, between 2012 and 2020 (data from the Study Centre for Perinatal Epidemiology SPE), whether conceived naturally, after using hormonal stimulation or assisted reproduction (IVF related procedures).

## 2. Methods and Materials

### 2.1. The Simplified IVF Culture System (SCS)

A detailed description of the simplified IVF culture system was described previously [7]. Briefly, SCS is composed of two standard plain glass tubes connected by catheter tubing. The first tube contains sodium bicarbonate and citric acid, which, after the addition of water, rapidly releases CO_2_ with considerable pressure that passes into the second culture tube containing the single-step culture medium. The atmosphere created with respect to CO_2_ and O_2_ concentration develops over several hours at 37 °C, and once the culture tube is disconnected, a stable pH between 7.29 and 7.35 is created. The conditions created remain unchanged with storage at normal refrigeration temperatures (10–15 °C). The atmosphere and pH in the stored tubes are stable for prolonged periods such that the culture medium could be equilibrated in advance of an IVF cycle. As SCS is an enclosed IVF system relaying on a self-contained, air-tight closed environment, changes in temperature, atmosphere or pH that could have adverse effects on developmental embryos are almost eliminated. The SCS system is consistent with human IVF, provided that the tube is kept at 37 °C during fertilisation and further embryo culture. Only a uniform temperature is necessary, which can be maintained quite simply and without the need for conventional and expensive incubators.

The reader is referred to the papers describing the SCS system in more detail [7,8].

### 2.2. Perinatal Outcome: Preterm Birth and Low Birth weight

Due to the nature of the SPE registration, it was impossible to distinguish between babies born after transfer of fresh or frozen embryos. Therefore, we compared our 175 SCS babies (fresh and frozen) with all registered SPE singletons born in Flanders between 2012 and 2020, excluding the SPE-registered SCS babies.

#### 2.2.1. SCS Data

All SCS singletons are part of a prospective study performed at the ZOL Hospitals in Genk, Belgium. The methodology of this study was described before [8,9]. All women suffered from unexplained infertility, tubal occlusion, or mild-to-moderate endometriosis. Severe male infertility cases with an Inseminating Motile Count (IMC) below 1 million were excluded and treated by means of ICSI.

All babies born after the transfer of SCS fresh and cryo/thawing embryos were prospectively studied. Three months after the expected day of delivery, patients were contacted by phone from our study nurse to inquire about obstetrical and perinatal outcomes of the newborn(s). The data obtained by phone were always compared with the information we received from the obstetrical unit where the delivery took place and adjusted if necessary.

In our prospective cohort study, we only observed 11 twin pregnancies (22 babies), and for this reason, only the 175 singletons were examined and statistically compared with the SPE-registered singletons.

#### 2.2.2. SPE Data

Since 1986 the SPE perinatal registry (www.zorg-en-gezondheid.be) systematically collects data on the medical, obstetrical, and perinatal events of each birth in Flanders of babies weighing ≥500 g birth weight, or gestational age ≥22 weeks if birth weight is unknown. The registry captures individual-level data abstracted from birth certificates and from obstetrical and neonatal medical records in the early postnatal and neonatal periods. Data are subjected to an error detection program, and checked for accuracy and completeness with extreme (considered outliers) or missing values confirmed with individual maternity units where appropriate. Correction and completion are ensured by telephone calls, additional data correction forms sent to midwives, obstetricians, and paediatricians, and visits to local departments if necessary. This results in a yearly global report and individual report for each centre. These data include pregnancies following natural conception, after assisted reproduction, including ovarian stimulation with or without IUI (Intrauterine Insemination) and IVF/ICSI procedures.

The SPE’s registered births in Flanders account for 52% of all births in Belgium. Data on births in the other Belgian regions (Brussels Capital Region and Walloon Region) account for 48% of the registered births and are collected by the Centre d’Épidémiologie Périnatale (CEpiP).

The SPE registers characteristics of the mother (age, parity, antecedents of caesarean section, BMI, and presence of hypertension and/or diabetes), the childbirth (type of onset of labour and mode of delivery), the child (gender, gestational age, birth weight, neonatal morbidity, and perinatal mortality) and more. In this particular study, we only use the mother’s age, the child’s gender, gestational age and birth weight, and the mode of conception (spontaneous, hormonal treatment, conventional IVF without ICSI, ICSI, and SCS).

### 2.3. Ethical Committee Approval

The prospective non-inferiority clinical trial comparing the effectiveness and perinatal outcome of SCS versus IVF/ICSI treatment cycles was approved by the Ethical Committees of Genk and the Free University of Brussels (reference no. 2011/011, approved 19 May 2011) and registered as B.U.N. 143201110348. The analysis comparing the cycle and perinatal outcome parameters of 175 SCS singletons and all singletons in Flanders during the period 2012–2020 was approved by the scientific committee of the SPE. The scientific committee granted approval for the analysis of the anonymised data. This study was exempt from approval by an Institutional Review Board because data were used for scientific purposes only.

### 2.4. Statistics

Data were imported into SPSS27 for initial data cleaning and data manipulation. All statistical analyses were conducted with R software version 4.0.3. Simple descriptive statistics were analysed, and group differences in the variable of interest were computed using a (post hoc) chi-square test. Continuous variables were compared by using a one-way ANOVA. Levene’s Test was used to check for homogeneity of variance, which led to the use of Welch’s *t*-test statistic, as equal variances could not be assumed. Scheffe and Tukey were used as post hoc tests. We performed two logistic regressions, one for each outcome variable of interest (low birth weight and preterm birth), with all predictors included in each model. The predictors included the child’s gender, mother’s age, and gestational age or weight at birth as continuous variables, depending on the outcome variable. To ensure there was no multicollinearity, we checked the correlations between all variables, which were found to be below 0.60. The mode of conception was added as a moderator in all possible associations, but no significant results were found (*p* > 0.05), which is why only the main effects are reported in the results section. Finally, the odds ratios were calculated with their 95% confidence intervals (CI).

## 3. Results

As shown in Table 1, we found a significant difference in the mother’s age, with younger mothers in spontaneous pregnancies and older mothers in the IVF without ICSI, ICSI and SCS groups. The average age of the mother at birth was significantly higher for IVF, ICSI and SCS compared to hormonal treatment. Additionally, the average age was also significantly lower for spontaneous pregnancies compared to hormonal treatment, IVF, ICSI and SCS. No significant difference was observed in the gender of the newborns between the different groups.

No significant difference in the average gestational age was observed for the SCS group with any of the other groups, except IVF, which had a significantly lower average gestational age compared to SCS. When compared to spontaneous pregnancies, the proportion of preterm (<37 weeks) births was significantly higher in the case of hormonal treatment, followed by IVF and ICSI. There was no significant difference in the proportion of PTB between SCS and any of the other groups.

No significant difference was found between spontaneous pregnancies, hormonal treatment, IVF, and ICSI concerning the average birth weight. A significant difference in average birth weight was found between SCS and IVF, ICSI and hormonal treatment, with a significantly higher average birth weight in the SCS group. There was no significant difference in the proportion of babies weighing less than 2500 g between SCS and any of the other groups. However, spontaneous pregnancies and hormonal treatments had a significantly lower proportion of newborns that weighed less than 2500 g compared to IVF or ICSI.

All main effects had a statistically significant impact on the outcome in our logistic regression analysis, as indicated by the likelihood ratio test (LRT) with *p*< 0.05 (Table 2). The risk of delivering a newborn before 37 weeks was significantly lower for girls and significantly higher for each unit increase in the mother’s age. On the other hand, the risk of delivering a newborn weighing less than 2500 g was significantly higher for girls and significantly lower for each unit increase in the mother’s age. As expected, the birth weight was a good predictor for gestational age and vice versa, with a higher birth weight leading to a lower risk of PTB and a higher gestational age leading to a lower risk of LBW. While the type of pregnancy improved the model significantly, we could not find a significant difference between SCS and any of the other methods.

**Figure 1:**
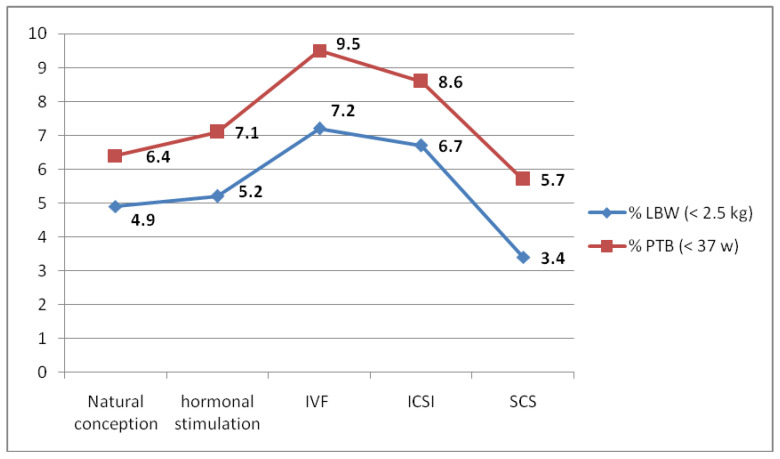
Comparison of preterm birth (<37 weeks, %) and low birth weight (<2.5 kg, %) between 175 babies born after SCS (simplified IVF culture system) and all babies born after natural conception (*n* = 516,252), hormonal treatment (*n* = 13,398), conventional IVF (*n* = 13,097) and ICSI (*n* = 10,943) in Flanders between 2012–2020 (red = figures for PTB, blue = figures for LBW).

Figure 1 provides a visual representation of the distinctions in PTB and LBW among the five investigated groups, offering valuable insights into the potential variations in health outcomes for each group.

We noted one perinatal mortality after fresh embryo transfer caused by an abruption placenta at 28 weeks gestation. The baby died immediately after birth; no clear reason could be found. We also observed one congenital malformation after the transfer of a cryo-thawed embryo. Although both cases were included in our sample, we were unable to conduct a reliable statistical analysis on these variables due to their limited occurrence (*n* = 1 each) in the SCS group. Therefore, we did not further investigate these variables as potential outcomes in our study. Nonetheless, we wish to report these cases as part of our findings.

## 4. Discussion

Infertility treatment is largely inaccessible to many people in LMICs. Even though assisted reproductive technologies have been used for over four decades, ART remains either unavailable or inaccessible to most people in resource-poor settings [23,24,25]. Direct medical costs paid by patients are significantly higher than the annual average income and GDP per capita, pointing to unaffordability and lack of access [26].

To overcome this barrier, the cost associated with ART needs to be reduced and requires the development of low-cost protocols and techniques.

From the beginning, the Walking Egg non-profit organisation aimed to initiate research projects to develop low-cost infertility treatment options resulting in the development of a low-cost, simplified IVF system (SCS) [6,7]. It turned out to be an effective and reliable method with pregnancy rates similar to conventional IVF [7,8].

In addition to its effectiveness, safety is the most important issue when introducing a new method of assisted reproduction. Previous reports have shown that the adverse outcome for babies born after using assisted reproductive technologies cannot be explained solely by the high prevalence of multiples. Additionally, singletons born after ART are more likely to have a poorer perinatal outcome when compared to naturally conceived singletons [16,17,27,28,29]. Fresh embryo transfer is associated with low birth weight and preterm birth, while frozen embryo transfer is associated with large-for-gestational-age babies [28].

We recently reported a favourable perinatal outcome for SCS singletons born after fresh embryo transfer when compared to singletons born after conventional IVF in Belgium [22]. The low prevalence of PTB and LBW we observed after using SCS prompted us to initiate a study to compare our results with a large cohort of naturally conceived newborns. Therefore, we used the SPE perinatal registry, which systematically collects data on the medical, obstetrical, and perinatal events of each birth in Flanders, Belgium, since 1986.

In this study comparing perinatal parameters between SCS singletons and all singletons born in Flanders during the same study period, it turned out that singletons born after using SCS, contrary to IVF and ICSI, did not differ from singletons born after natural conception in its proportion of preterm births, low birth weight rate and average birth weight. We also observed a significantly lower average birth weight for newborns born after hormonal stimulation and IVF/ICSI when compared to SCS babies. Above this, significantly more children weighing under 2500 g were observed in the IVF or ICSI group compared to SCS. Considering the safety of the simplified IVF culture system, the reassuring results obtained in this study confirm the findings of our previous report in which we also found significantly less babies weighing below 2500 g when compared to all IVF babies born in Belgium during the same study period [22].

To explain this low prevalence of preterm birth and low birth weight for SCS babies, very similar to the figures obtained for naturally conceived babies, we postulate that our enclosed SCS environment closely imitates natural conception with temperature control as the only variable. Epigenetic reprogramming is susceptible to environmental changes and non-physiological conditions, such as those applied during in vitro culture, including changes in pH and temperature, oxygen tension, intracytoplasmic sperm injection, and cryopreservation of embryos [30,31]. It is possible that our perinatal outcome results might be secondary to less pronounced epigenetic modifications and dysregulation of the embryos compared to regular IVF or ICSI, but this is still hypothetical and unproven. More studies are needed to elucidate the mechanisms by which IVF may contribute to adverse outcomes, with special attention to the role of cryopreservation and extended culture of human embryos in different environments caused by different culture media and culture conditions [27,30].

A major strength of this study is that all SCS data were registered prospectively with a meticulous follow-up. Historically, SPE data are also considered very reliable, with regular feedback about missing data, errors, and inconsistencies.

The most important limitation of this study is the low number of SCS babies when compared to the large group of SPE-registered singletons. Therefore, the results of this study should be interpreted with caution as the different groups (mode of conception) are very different. The absence of significant differences between the SCS group and the other groups is not surprising because the sample size is only 175. The lack of significant differences observed in this study is more likely due to a lack of sensitiveness (too different sample sizes) than due to a real non-difference. To address this limitation, we suggest using bootstrapping as a resampling technique to estimate the variability and distribution of the statistical parameters of interest, which could improve the reliability and validity of the analysis.

Although we adjusted for females’ age and gender of the newborn when comparing the different groups, many residual confounding factors cannot be excluded, in particular, related to duration and cause of infertility or whether fresh or frozen embryos were transferred. All these parameters are important when studying perinatal outcomes, but we could not adjust for the parameters because these data were not available in the SPE registration, another limitation of this study. We recommend incorporating additional confounding factors in future analysis, such as the mother’s BMI, presence of hypertension and/or diabetes, and socio-demographic variables, all of which are available in the SPE registration.

On the other hand, our first aim was to compare our SCS singletons with a large series of naturally conceived singletons, being aware of the lack of some important data in both registries and the small number of SCS newborns. Taking into account all these limitations, we still observed a low PTB and LBW for SCS singletons, similar to figures published for singletons born after natural conception.

## 5. Conclusions

Due to the unexpectedly low prevalence of PTB and LBW for SCS babies reported in a previous study, we aimed to compare the perinatal outcome of SCS singletons and a large cohort of naturally conceived (NC) singletons. Our results show comparable PTB and LBW rates for SCS and NC singletons. This strengthens our belief that the SCS-IVF technology can be used as a safe method with an excellent perinatal outcome. Because of the low costs associated with SCS, it opens perspectives to increase the general applicability of assisted reproductive techniques in areas where conventional IVF laboratory costs are the limiting factor to have access to complex fertility treatments.

## Figures and Tables

**Table 1:** Comparison of mother’s age and different perinatal outcome parameters between 175 babies born after SCS and all babies born after natural conception, hormonal treatment, conventional IVF and ICSI in Flanders between 2012–2020 (*n* = 553,865).

	Spontaneous (*n* = 516,252; 93%) *n* (%)	Hormonal Treatment (*n* = 13,398; 2%) *n* (%)	IVF (without ICSI) (*n* = 13,097; 2%) *n* (%)	ICSI (*n* = 10,943; 2%) *n* (%)	SCS (*n* = 175; 0%) *n* (%)	*p*-Value
**Mother’s age** [mean (SD)]	30.25 (4.74) ^a^	30.99 (4.20) ^b^	33.47 (4.71) ^c^	33.37 (4.58) ^c^	33.60 (4.28) ^c^	<0.001 *
**Child’s gender** [*n* (%)]						
Male	264,904 (51.3) ^a^	6784 (50.6) ^a^	6629 (50.6) ^a^	5528 (50.5) ^a^	104 (59.4) ^a^	0.018
Female	251,344 (48.7) ^a^	6614 (49.4) ^a^	6468 (49.4) ^a^	5415 (49.5) ^a^	71 (40.6) ^a^	
**Gestational age** [mean (SD)]	38.84 (1.81) ^a^	38.76 (1.89) ^a,b^	38.57 (2.19) ^b^	38.65 (2.15) ^a,b^	38.85 (1.82) ^a^	<0.001 *
<37 weeks [*n* (%)]	33,059 (6.4) ^a^	949 (7.1) ^b^	1241 (9.5) ^c^	941 (8.6) ^c^	10 (5.7) ^a,b,c^	<0.001
≥37 weeks [*n* (%)]	483,187 (93.6) ^a^	12,449 (92.9) ^b^	11,856 (90.5) ^c^	10,002 (91.4) ^c^	165 (94.3) ^a,b,c^	
**Birth weight** [mean (SD)]	3349.46 (532.92) ^a,b^	3328.63 (539.17) ^a^	3300.23 (593.84) ^a^	3319.72 (586.21) ^a^	3413.26 (559.31) ^b^	<0.001 *
<2500 g [*n* (%)]	25,338 (4.9) ^a^	693 (5.2) ^a^	940 (7.2) ^b^	738 (6.7) ^b^	6 (3.4) ^a,b^	<0.001
≥2500 g [*n* (%)]	490,914 (95.1) ^a^	12,705 (94.8) ^a^	12,157 (92.8) ^b^	10,205 (93.3) ^b^	169 (96.6) ^a,b^	

* One-way ANOVA with Welch statistic. ^a,b,c^ Superscripts (a, b, and c) indicate significant differences between proportions of the same variable category across different types of pregnancies (spontaneous, hormonal treatment, IVF, ICSI, and SCS). Cells from the same row with the same superscript do not differ significantly, while cells from the same row with different superscripts indicate significant differences in proportions (nominal variables: post hoc *X*^2^-test *p* > 0.05; and continuous variables: post hoc Scheffe test *p* > 0.05). Bold formatting is employed in the table to distinguish variable names from their corresponding categories, enhancing clarity and preventing confusion. Notes: As the comparisons in this table involved 6 independent tests, we adopted a Bonferroni-corrected significance level of 0.05/6 = 0.008 for these analyses. Abbreviations: IVF = In Vitro Fertilization; ICSI = Intra Cytoplasmic Sperm Injection; SCS = Simplified IVF Culture System; SD = Standard Deviation.

**Table 2:** Logistic regression analyses for the association between the mode of conception, the child’s gender and the mother’s age and preterm birth and low birth weight (*n* = 553,865).

Predictors	PTB *			LBW *		
	Odds Ratio	95% CI Odds Ratio	*p*-Value (LRT)	Odds Ratio	95% CI Odds Ratio	*p*-Value (LRT)
**Pregnancy** (ref. SCS)			<0.001			<0.001
Spontaneous	1.00	0.46–2.50		1.46	0.53–5.90	
Hormonal treatment	1.07	0.49–2.68		1.39	0.50–5.62	
IVF (without ICSI)	1.22	0.56–3.08		1.62	0.59–6.58	
ICSI	1.16	0.53–2.92		1.65	0.60–6.69	
**Child’s gender** (ref. Male)			<0.001			<0.001
Female	0.53	0.51–0.54		1.80	1.74–1.86	
**Mother’s age**	1.01	1.01–1.02	0.011	0.99	0.98–0.99	<0.001
**Birth weight** (in grams)	0.99	0.99–0.99	<0.001	-	-	-
**Gestational age** (in weeks)	-	-	-	0.34	0.34–0.35	<0.001

* PTB was coded as gestational age ‘<37 weeks (1)’ and ‘≥37 weeks (0)’ and LBW as birth weight ‘<2500 g (1)’ and ‘≥2500 g (0)’. Bold formatting is employed in the table to distinguish variable names from their corresponding categories, enhancing clarity and preventing confusion. Abbreviations: PTB = Preterm Birth; LBW = Low Birth Weight; CI = Confidence Interval; LRT = Likelihood Ratio Test; SCS = Simplified IVF Culture System; IVF = In Vitro Fertilization; ICSI = Intra Cytoplasmic Sperm Injection.

## Data Availability

All data generated or analysed in this study are included in this published article.

## References

[1] Steptoe P.C., Edwards R.G. (1978). Birth after the reimplantation of a human embryo. Lancet.

[2] Ombelet W., Cooke I., Dyer S., Devroey P. (2008). Infertility and the provision of infertility medical services in developing countries. Hum. Reprod. Update.

[3] Inhorn M.C., Patrizio P. (2015). Infertility around the globe: New thinking on gender, reproductive technologies and global movements in the 21st century. Hum. Reprod. Update.

[4] Chiware T.M., Vermeulen N., Blondeel K., Farquharson R., Kiarie J., Lundin K., Matsaseng T.C., Ombelet W., Toskin I. (2021). IVF and other ART in low- and middle-income countries: A systematic landscape analysis. Hum. Reprod. Update.

[5] Afferri A., Allen H., Booth A., Dierickx S., Pacey A., Balen J. (2022). Barriers and facilitators for the inclusion of fertility care in reproductive health policies in Africa: A qualitative evidence synthesis. Hum. Reprod. Update.

[6] Ombelet W. (2014). Is global access to infertility care realistic? The Walking Egg Project. Reprod. Biomed. Online.

[7] Van Blerkom J., Ombelet W., Klerkx E., Janssen M., Dhont N., Nargund G., Campo R. (2014). First Births with a Simplified Culture System for Clinical IVF and ET. Reprod. Biomed. Online.

[8] Ombelet W., Van Blerkom J., Nargund G., Van der Auwera I., Janssen M., Dhont N., Bosmans E., Boshoff G., Vertessen V.J., Campo R. (2022). Multiyear outcomes using sibling oocytes demonstrates safety and efficacy of a simplified culture system consistent with use in a low-cost IVF setting. Reprod. Biomed. Online.

[9] Ombelet W., Van Blerkom J., Nargund G., Janssen M., Jacobs P., Van der Auwera I., Dhont N., Bosmans E., Vertessen V.J., Campo R. (2022). Perinatal outcome of babies born after using a simplified culture system for IVF versus ICSI followed by conventional culturing with sibling oocytes: A multi-year prospective cohort study. Reprod. Biomed. Online.

[10] Chawanpaiboon S., Vogel J.P., Moller A.B., Lumbiganon P., Petzold M., Hogan D., Landoulsi S., Jampathong N., Kongwattanakul K., Laopaiboon M. (2019). Global, regional, and national estimates of levels of preterm birth in 2014: A systematic review and modelling analysis. Lancet Glob. Health.

[11] Lee A.C., Blencowe H., Lawn J.E. (2019). Small babies, big numbers: Global estimates of preterm birth. Lancet Glob. Health.

[12] Vogel J.P., Chawanpaiboon S., Moller A.B., Watananirun K., Bonet M., Lumbiganon P. (2018). The global epidemiology of preterm birth. Best Pract. Res. Clin. Obstet. Gynaecol..

[13] Wennerholm U.B., Bergh C. (2020). Perinatal outcome in children born after assisted reproductive technologies. Upsala J. Med. Sci..

[14] Li Z., Wang Y.A., Ledger W., Sullivan E.A. (2014). Birthweight percentiles by gestational age for births following assisted reproductive technology in Australia and New Zealand, 2002–2010. Hum. Reprod..

[15] Ombelet W., Martens G., Bruckers L. (2016). Pregnant after assisted reproduction: A risk pregnancy is born! 18-years perinatal outcome results from a population-based registry in Flanders, Belgium. Facts Views Vis. Obgyn.

[16] Qin J.B., Sheng X.Q., Wu D., Gao S.Y., You Y.P., Yang T.B., Wang H. (2017). Worldwide prevalence of adverse pregnancy outcomes among singleton pregnancies after in vitro fertilization/intracytoplasmic sperm injection: A systematic review and meta-analysis. Arch. Gynecol. Obstet..

[17] Cavoretto P., Candiani M., Giorgione V., Inversetti A., Abu-Saba M.M., Tiberio F., Sigismondi C., Farina A. (2018). Risk of spontaneous preterm birth in singleton pregnancies conceived after IVF/ICSI treatment: Meta-analysis of cohort studies. Ultrasound Obstet. Gynecol..

[18] Sunderam S., Kissin D.M., Zhang Y., Jewett A., Boulet S.L., Warner L., Kroelinger C.D., Barfield W.D. (2022). Assisted Reproductive Technology Surveillance—United States, 2018. MMWR Surveill. Summ..

[19] Berntsen S., Pinborg A. (2018). Large for gestational age and macrosomia in singletons born after frozen/thawed embryo transfer (FET) in assisted reproductive technology (ART). Birth Defects Res..

[20] Elias F.T.S., Weber-Adrian D., Pudwell J., Carter J., Walker M., Gaudet L., Smith G., Velez M.P. (2020). Neonatal outcomes in singleton pregnancies conceived by fresh or frozen embryo transfer compared to spontaneous conceptions: A systematic review and meta-analysis. Arch. Gynecol. Obstet..

[21] Terho A.M., Pelkonen S., Opdahl S., Romundstad L.B., Bergh C., Wennerholm U.B., Henningsen A.A., Pinborg A., Gissler M., Tiitinen A. (2021). High birth weight and large-for-gestational-age in singletons born after frozen compared to fresh embryo transfer, by gestational week: A Nordic register study from the CoNARTaS group. Hum. Reprod..

[22] Ombelet W., Van Blerkom J., Bruckers L., Dhont N., Nargund G., Campo R. (2023). Promising Perinatal Outcome After Using a Simplified Low-Cost IVF Culture System Specifically Designed for Resource Poor Countries. J. Clin. Med..

[23] Botha B., Shamley D., Dyer S. (2018). Availability, effectiveness and safety of ART in sub-Saharan Africa: A systematic review. Hum. Reprod. Open.

[24] Dyer S.J., Patel M. (2012). The economic impact of infertility on women in developing countries—A systematic review. Facts Views Vis. ObGyn.

[25] Dyer S.J., Sherwood K., McIntyre D., Ataguba J.E. (2013). Catastrophic payment for assisted reproduction techniques with conventional ovarian stimulation in the public health sector of South Africa: Frequency and coping strategies. Hum. Reprod..

[26] Njagi P., Groot W., Arsenijevic J., Dyer S., Mburu G., Kiarie J. (2023). Financial costs of assisted reproductive technology for patients in low- and middle-income countries: A systematic review. Hum. Reprod. Open.

[27] Pinborg A., Wennerholm U.B., Romundstad L.B., Loft A., Aittomaki K., Söderström-Anttila V., Nygren K.G., Hazekamp J., Bergh C. (2013). Why do singletons conceived after assisted reproduction technology have adverse perinatal outcome? Systematic review and meta-analysis. Hum. Reprod. Update.

[28] Berntsen S., Söderström-Anttila V., Wennerholm U.-B., Laivuori H., Loft A., Oldereid N.B., Romundstad L.B., Bergh C., Pinborg A. (2019). The health of children conceived by ART: ‘the chicken or the egg?’. Hum. Reprod. Update.

[29] Pandey S., Shetty A., Hamilton M., Bhattacharya S., Maheshwari A. (2012). Obstetric and perinatal outcomes in singleton pregnancies resulting from IVF/ICSI: A systematic review and meta-analysis. Hum. Reprod. Update.

[30] Sciorio R., El Hajj N. (2022). Epigenetic Risks of Medically Assisted Reproduction. J. Clin. Med..

[31] Sciorio R., Tramontano L., Rapalini E., Bellaminutti S., Bulletti F.M., D’Amato A., Manna C., Palagiano A., Bulletti C., Esteves S.C. (2023). Risk of genetic and epigenetic alteration in children conceived following ART: Is it time to return to nature whenever possible?. Clin. Genet..

